# Reversible Behavioral Deficits in Rats during a Cycle of Demyelination-Remyelination of the Fimbria

**DOI:** 10.1371/journal.pone.0053775

**Published:** 2013-01-22

**Authors:** Natalia M. Grin'kina, Samah G. Abdel-Baki, Peter J. Bergold

**Affiliations:** 1 Robert F. Furchgott Center for Neural Science, State University of New York Downstate Medical Center, Brooklyn, New York, United States of America; 2 Department of Physiology, Pharmacology and Neurology, State University of New York Downstate Medical Center, Brooklyn, New York, United States of America; University of Pittsburgh, United States of America

## Abstract

Traumatic brain injury (TBI) selectively damages white matter. White matter damage does not produce deficits in many behavioral tests used to analyze experimental TBI. Rats were impaired on an active place avoidance task following inactivation of one hippocampal injection of tetrodotoxin. The need for both hippocampi suggests that acquisition of the active place avoidance task may require interhippocampal communication. The controlled cortical impact model of TBI demyelinates midline white matter and impairs rats on the active place avoidance task. One white matter region that is demyelinated is the fimbria that contains hippocampal commissural fibers. We therefore tested whether demyelination of the fimbria produces deficits in active place avoidance. Lysophosphatidylcholine (LPC) was injected stereotaxically to produce a cycle of demyelination-remyelination of the fimbria. At 4 days, myelin loss was observed in the fimbria of LPC-, but not saline-injected rats. Fourteen days after injection, myelin content increased in LPC-, but not saline-injected rats. Three days after injection, both saline- and LPC-injected rats had similar performance on an open field and passive place avoidance task in which the rat avoided a stationary shock zone on a stationary arena. The following day, on the active place avoidance task, LPC-injected rats had a significantly higher number of shock zone entrances suggesting learning was impaired. At 14 days after injection, saline- and LPC-injected rats had similar performance on open field and passive place avoidance. On active place avoidance, however, saline- and LPC-injected rats had a similar number of total entrances suggesting that the impairment seen at 4 days was no longer present at 14 days. These data suggest that active place avoidance is highly sensitive to white matter injury.

## Introduction

Traumatic brain injury damages both grey and white matter but there is a selective damage to white matter. White matter is not uniformly vulnerable since longitudinal and commissural white matter tracts are selectively injured [1]. TBI also produces cognition and memory deficits [2]. These deficits are seen using animal models of TBI using the Barnes maze, Morris water maze, and the radial arm maze when testing in an animal model of TBI using an injury intensity that damages both grey and white matter [2], [3], [4], [5], [6], [7]. Damage only to white matter does not produce deficits in these tasks. Mice acquired and retained the Barnes Maze despite unilateral lesions of the corpus callosum and ventral hippocampal commissure [8], [9]. Rats acquired the Morris water maze after inactivation of one hippocampus with lidocaine or tetrodotoxin or after bilateral severing of the perforant pathway [9], [10], [11]. Unilateral lesions of the hippocampus or lesion of the hippocampal commissures did not impair acquisition of the radial arm maze [12]. These data suggest that acquisition of hippocampus-dependent spatial tasks such as Barnes maze, water, and radial arm mazes occurs when only grey matter is damaged and does not require communication between the two hippocamppi.

The active place avoidance task may be more sensitive to white matter injury than the Barnes maze, Morris water maze and radial arm mazes. Unlike purely spatial tasks such as Morris water maze, the active place avoidance task requires the sensory segregation of relevant and irrelevant cues to acquire the location of a stationary shock zone. Rats learn to avoid the shock zone by attending to distal visual cues in the room while disregarding the irrelevant olfactory cues on the arena [13]. Naïve rats do not learn the shock zone location when one hippocampus is inactivated with tetrodotoxin, and the inactivation even impairs avoiding a familiar shock zone [14]. The unilateral tetrodotoxin injection also altered neuronal discharge in the uninjected hippocampus by co-activating pyramidal cell pairs that previously discharged independently [15]. Thus, proper communication between the two hippocamppi may be needed for acquisition and performance of the active place avoidance task. A need to coordinate the activity of the two hippocamppi may underlie a greater sensitivity of the active place avoidance task to white matter injury than other commonly used behavioral tasks.

In a moderate version of controlled cortical impact model of TBI, rats learn the location of a stationary shock zone, but have a long-lasting impairment when the arena rotates [16]. Injured rats can acquire the shock zone location following treatment with the drug combination of minocycline and N-acetylcysteine [17]. Moderate CCI damages both grey and white matter [16], [17]. Damage to grey matter after CCI is located predominantly at the impact site [17]. White matter damage, in contrast, is diffuse with most injury located at the medial aspect of midline white matter structures such as the corpus callosum and the fimbria [17], [18]. MINO plus NAC had no effect on the volume lost at the impact site, yet prevented myelin loss suggesting that the drugs selectively prevented white matter injury.

This study tested whether acquisition of the active place avoidance task is affected by a white matter injury that is more localized than the injury produced by CCI. A dilute lysophosphatidylcholine (LPC) solution injected stereotaxically into white matter produces a limited neuroinflammation and a single synchronous cycle of demyelination and remyelination [19]. In rats, LPC injection induces a demyelination of the corpus callosum within days that is repaired by remyelination within 2 weeks [20]. Oligodendrocytes are partially spared resulting in a spontaneous and synchronous remyelination [19], [20], [21]. Axonal loss is limited due to the rapid induction of demyelination and remyelination [19]. LPC demyelinated the corpus callosum 4 days after injection [20]. CCI induces myelin loss in multiple white matter tracts including the fimbria [17]. CCI also produces behavioral deficits in the active place avoidance task [16], [17].

The fimbria contains commissural fibers that project via the ventral hippocampal commissure and makes homotypic and heterotypic connections between the CA1, CA2 and CA3 regions [22]. The fimbria also contains fibers that project via the fornix to multiple brain regions [22]. We therefore injected LPC into the fimbria to determine whether a purely white matter injury can impair acquisition of the active place avoidance task.

## Materials and Methods

### LPC injections

Upon arrival at SUNY-Downstate, Sprague-Dawley rats (250–300 g, Charles River, Wilmington, MA) were placed on an inverted day-night cycle for one week. Rats were then deeply anesthetized using isoflurane (3–5%) in oxygen (0.8 L/min). A 10 µl Hamilton Syringe was used to inject 0.3 µl LPC (1% (v/v) in saline) over a period of 5 minutes into the fimbria (ML- 0.5, AP - (-1.5), DV - 4) [23]. The needle was retained in the brain for 5 minutes and then removed. Control rats received a saline injection at the same coordinates. Following completion of the injection, the syringe was retained an additional 4 minutes to complete the diffusion of liquid from the needle tip. After removal of the syringe, the incision was sutured and the animals recover in their home cages for 4 or 14 days. This study was carried out in strict accordance with the recommendations in the Guide for the Care and Use of Laboratory Animals of the National Institutes of Health. This study has been approved by the Institutional Animal Care and Use Committee at the State University of New York-Downstate Medical Center (Protocol ID: 08-477-10). All efforts were made to minimize animals' suffering.

### Behavioral testing

The rats received the hierarchy of three behavior tasks slightly modified from Abdel-Baki, et al. 2009. These behavioral tests have been previously developed to detect cognitive deficits after experimental TBI in rodents [16]. Rats received a stereotaxic injection of either saline or LPC and returned to their home cages. Either 3 or 13 days after injection, rats were subjected to open field and passive place avoidance. The first task measured the total distance traveled in a 10-minute open field test of a stationary arena. The open field test examined the innate ability of the rat to explore a novel environment. Immediately after the open field test, rats received four 10-minute trials of passive place avoidance in which a 60° shock zone was added to the arena previously used for the open field test. On the 1^st^ trial of passive place avoidance, the shock intensity was titrated. Shock intensity was set at 0.2 mA and increased by 0.1 mA increments until the rat was induced to leave the shock zone. The total distance traveled and number of shock zone entrances was also assessed during passive place avoidance. The number of shock zone entrances measured the ability to avoid the shock zone and the total distance traveled measured locomotion. The following day, rats were tested on active place avoidance in which the arena rotated one revolution per minute while the same shock zone used in passive avoidance remained stationary. In passive place avoidance, rats avoid the arena using either olfactory cues placed on the arena or distal visual cues in the room. During active place avoidance, rats avoid the shock zone by ignoring irrelevant olfactory cues deposited on the arena and attend to prominent visual cues in the room [13]. Active place avoidance was tested using 6 ten-minute trials separated with a 10-minute intertrial interval. The parameters assessed during active place avoidance were shock zone entrances, time to first entrance. Speed, linearity and distance traveled were also assessed. Shock zone entrances assayed the ability of the rat to learn the shock zone location, while time to first entrance assayed the ability of the rat to retain the shock zone location from the previous trial. Speed was assayed every 2 s and averaged over the 10 minute trial. Linearity was determined every 2 s and is average of the ratio of distance_linear_ and distance_integrated_, where distance_integrated_ is the sum of the distances moved each 33 ms in a 2-second interval, and distance_linear_ is the linear distance between the locations at the start and end of the 2-second interval. Since the intensity of shock needed for a rat to leave the shock zone was determined during passive place avoidance, number of shocks per entrances assessed the motivation to escape shock during active place avoidance.

### Histology

**Figure 1:**
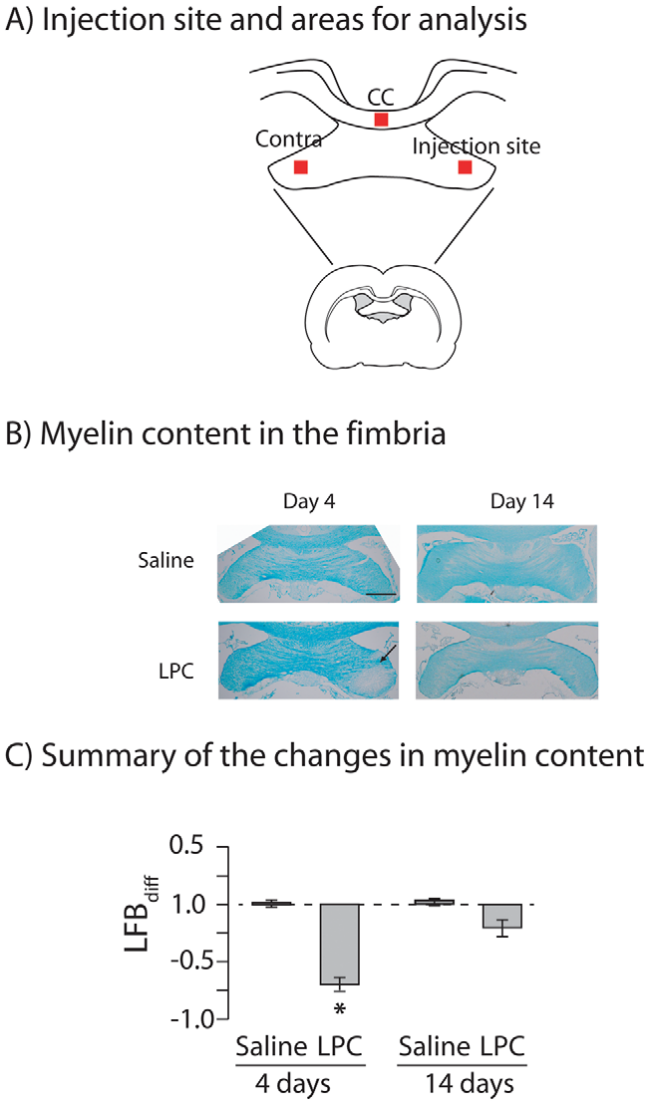
LPC produces a reversible demyelination in the fimbria. **Panel A,** A schematic diagram of a coronal section of rat brain located −1.08 mm from Bregma. The injection site and regions analyzed for myelin content are indicated [23]. **Panel B,** Myelin in the ipsilateral and contralateral fimbria stained with luxol fast blue in saline and LPC-treated rats at 4 and 14 days after injection. An arrow points to an area of demyelination day 4 days after LPC injection. Scale bar, 1 mm. **Panel C.** Summary of the changes in myelin content. The difference in the myelin content in the ipsilateral and contralateral fimbria was normalized to luxol fast blue staining in the corpus callosum (LFB_diff_). There was a significant effect of days (F_(7,47)_  = 23.2, p<0.0001). Four days after injection, myelin content in the ipsilateral fimbria differed significantly in saline- and LPC-injected rats. At 4 days, there was a significant difference in myelin content between the ipsilateral and contralateral fimbria (*p<0.0001). This difference was no longer evident at day 14 (p>0.05). Myelin content significantly increased in the ipsilateral fimbria in LPC-injected rats between 4 and 14 days after injection (*p<0.0001). These data suggest that LPC produced a demyelination of the ipsilateral fimbria at 4 days that remyelinated at 14 days.

Four or 14 days after saline or LPC injection, rats were deeply anesthetized using isoflurane (3–5%) in oxygen (0.8 L/min) followed by transcardial infusion with paraformaldehyde (4% (w/v)). Coronal brain sections 1.08 mm rostral from Bregma were prepared containing the corpus callosum, ventral hippocampal commissure and fimbria (figure 1A). Sections were stained using Luxol fast blue according to the manufacturer's instructions (American Mastertech, Lodi, CA). Digital images were prepared and the amount of Luxol Fast Blue staining was assayed using Image J software in the two fimbria and the corpus callosum. To ensure that LFB staining was measuring myelin, dye intensity in the heavily myelinated fimbria and the corpus callosum was subtracted from the weakly myelinated paraventricular nucleus of the thalamus located on the ventral surface of the 3^rd^ ventricle. Differences in the amount of Luxol fast blue staining between the two fimbria was measured using the following formula: LFB_diff_  =  (LFB_ipsi_ – LFB_contra_/LFB_cc_) where LFB_ipsi_ and LFB_contra_ are the amounts of staining intensity in the ipsilateral and contralateral fimbria, LFB_cc_ is the staining intensity in the corpus callosum and LFB_diff_ is the normalized difference between the two fimbria.

### Statistical analysis

Group comparisons by ANOVA compared saline and LPC-injected groups. Student Newman-Keul's post-hoc tests were done as necessary. Statistical significance was set at 0.05 and all values are presented as mean ± SEM. In the histological analysis, luxol fast blue dye intensity at 4 and 14 days was assessed by one-way ANOVA followed pair wise post-hoc comparisons. On open field, distance traveled was assessed by one-way ANOVA. On passive place avoidance, the number of shock zone entrances was assayed by repeated two-way ANOVA on the factors of group and trial. Shock intensity at both 4 and 14 days was assayed by one-way ANOVA. On active place avoidance, the number of shock zone entrances was also assessed by repeated two-way ANOVA on the factors of group and trial. The remaining parameters measured during active place avoidance, shocks per entrance, distance traveled, average speed and linearity were evaluated using one-way ANOVA to compare the groups independently of the effects of trial.

## Results

### LPC injection produces a reversible localized, unilateral demyelination of the fimbria

Stereotaxic injections of LPC were used to examine the consequences of demyelination-remyelination of the fimbria in the absence of the more widespread white and grey matter damage produced by CCI [17]. LPC (n = 6) or saline (n = 6) was stereotaxically injected into the fimbria. Four days after saline or LPC injection, rats were sacrificed and coronal sections prepared containing the fimbria ipsilateral and contralateral to the injection site. The sections also contained ventral hippocampal commissure, and corpus callosum (Figure 1A, B). Myelin was stained with luxol fast blue. Myelin content in the fimbria ipsilateral to the LPC injection was significantly lower when compared to the contralateral fimbria (ANOVA, F_(7,39)_  = 23.29, p<0.001; post-hoc, p<0.001; Figure 1B, C). After saline injection, ipsilateral and contralateral fimbria had similar myelin content (post-hoc, p>0.05). Myelin content in the contralateral fimbria was similar after saline or LPC injection (post-hoc, p>0.05). These data suggest that LPC produced a localized demyelination of the ipsilateral fimbria 4 days after injection.

To test whether demyelination in the ipsilateral fimbria was followed by remyelination, a second set of rats received stereotaxic LPC or saline injections into one fimbria and myelin content was examined 14 days after injection. The ipsilateral and contralateral fimbria had similar myelin content 14 days after injection of either LPC or saline (post-hoc, p>0.05). Myelin content in the ipsilateral fimbria at 14 days after LPC injection was significantly increased when compared to myelin content at 4 days (post-hoc, p<0.001). In contrast, myelin content was similar in the contralateral fimbria at 4 or 14 days after LPC injection (post-hoc, p>0.05). These data strongly indicate that the localized demyelination of the ipsilateral fimbria at 4 days post-injection had spontaneously remyelinated by 14 days.

### Rats are impaired in the active place avoidance task 4 days after LPC injection

The behavior or rats could be assessed at times of demyelination and remyelination. Rats were divided into two groups that received a unilateral injection of either LPC (n = 6) or saline (n = 6) into the fimbria. Three days after injection, the two groups began a two-day examination of three behavioral tasks. On open field testing, both saline- and LPC-injected rats moved an equivalent distance suggesting both groups retained a similar ability to explore a new environment (F_(3,23)_  = 0.21, p = 0.88) (Table 1). Saline- or LPC-injected rats needed an equivalent amount of electrical current to exit the shock zone (F_(3,23)_  = 0.08, p = 0.97) (Table 1). This suggests that both saline and LPC rats had an equivalent ability to feel shock and exit the shock zone. In passive place avoidance, both the saline- and LPC-injected groups traveled an equivalent distance during passive place avoidance (F_(1,30)_  =  3.37, p = 0.09). LPC- and saline-injected groups both learned to avoid the shock zone since there was a significant effect of trial (F_(1,30)_  = 5.84, p<0.005), but not of group (F_(1,10)_  = 2.57, p>0.1). By the 4^th^ trial, both LPC-injected and saline-injected rats either entered the shock zone once or not at all. These data suggest that, saline and LPC-injected rats both learned to avoid the shock zone in passive place avoidance.

**Table 1:** Measurements of rat behavior during open field, passive place avoidance and active place avoidance.

Task	Parameter	Saline 4d	LPC 4d	Saline 14d	LPC 14d
Open Field	Total distance	23.63±3.51	23.3±6.69	24.19±4.91	28.24±3.91
Active Place Avoidance	Speed	4.86±0.34	3.60±0.62	4.40±0.24	4.13±0.20
Active Place Avoidance	Linearity	0.61±0.02	0.70±0.01	0.64±0.02	0.57±0.02
Active Place Avoidance	Time to first entrance	158.2±95.5	34.3±6.9	147.2±91.1	234.5±115.8
Active Place Avoidance	Shocks/Entrance	0.69±0.22	2.93±0.67*	1.81±0.11	0.83±0.48
Passive and active place avoidance	Shock intensity	0.33±0.05	0.033±0.036	0.35±0.04	0.30±0.05

**Figure 2:**
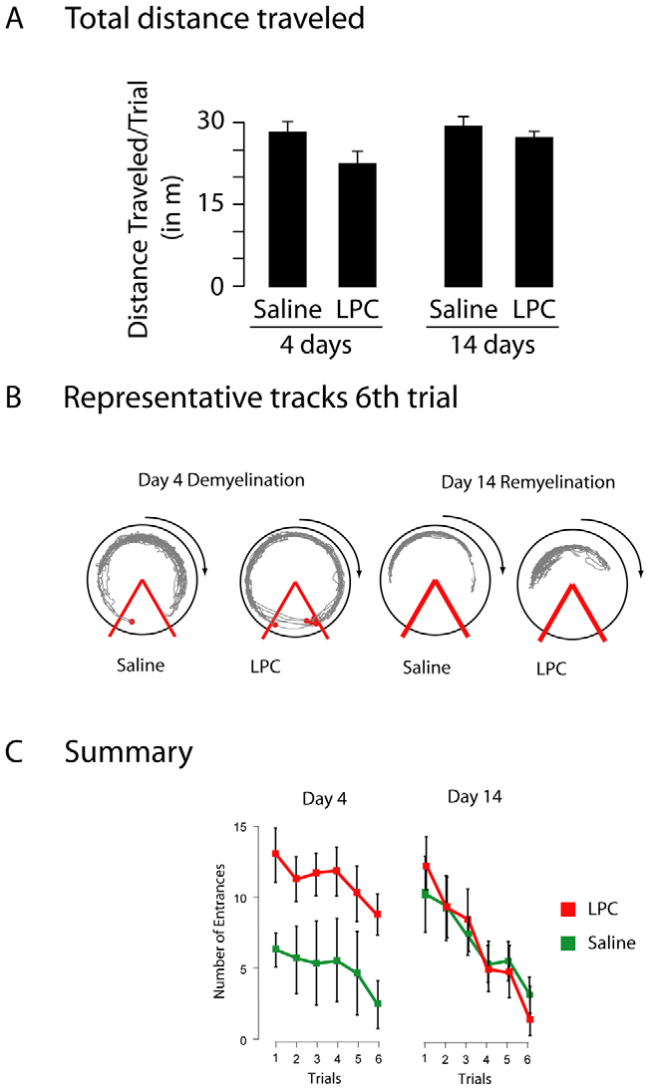
Behavioral analyses at times of demyelination and remyelination. **Panel A,** Total distance traveled during active place avoidance. Rats injected with LPC or saline traveled a similar distance at both 4 and 14 days (F_(3,19)_  = 1.71). **Panel B,** Representative tracks of rats 4 or 14 days after saline or LPC injection on the final (6^th^) trial of active place avoidance. Red lines indicate the shock zone boundaries and the red circles indicate the locations where shocks were delivered. **Panel C,** Summary of the number of shock zone entrances in each trial of active place avoidance. At 4 days, saline- and LPC-injected animals showed a significant effect of treatment (F_(1,10)_  = 16.31, p<0.005) and trial (F_(5,50)_  = 4.20, p<0.005) with no interaction of treatment and trial (F_(5,50)_  = 0.47). At 14 days, saline and LPC-injected animals had an no significant effect of treatment (F_(1,10)_  = 0.23), but there was a significant effect of trial (F_(5,55)_  = 5.81, p<0.001). Saline-treated animals analyzed at 4 and 14 days showed no significant effect of days (F_(1,12)_  = 1.78), but there was a significant effect of trial (F_(5,60)_  = 3.35, p<0.0005). LPC-injected rats at 4 and 14 days, trended toward an effect of days (F_(1,9)_  = 4.0, p = 0.08) but had a significant effect of trial (F_(5,45)_  = 10.4 p<0.0001) and a significant interaction of treatment and trial (F_(5,45)_  =  2.62, p<0.05). These data suggest that saline-injected animals acquired the active place avoidance task at both 4 and 14 days, whereas LPC-injected animals acquired the task only at 14 days. Saline- and LPC-injected animals differed in their acquisition the task at 4, but not 14 days.

The next day, rats were tested on active place avoidance. Saline- and LPC- injected rats traveled an equivalent distance during passive place avoidance training (Figure 2A). LPC-injected rats also did not differ in either speed or linearity (Table 1). By the 6th and final trial of active place avoidance, however, LPC-injected rats had multiple entrances into the shock zone as compared to saline-injected (Figure 2B). The differences between the number of shock zone entrances was confirmed by a significant group effect (F_(1,10)_  = 16.31, p<0.005). These differences were further supported by a significant trial effect (F_(5,50)_  = 14.2, p<0.005), but there was no interaction between the factors of group and trials (F_(5,50)_  = 0.42, p = 0.83). The LPC-injected group also had significantly more shocks per entrance as compared to the saline-injected group (Table 1). This difference was not due to the ability of LPC-injected rats to feel shock since the equivalent amount of current was needed to induce both groups to leave the shock zone. These data suggest that, 4 days after injection, the saline-injected group was able to lower the number of entrances and to learn the location of the shock zone while the LPC-injected group was impaired.

### Fourteen days after injection, LPC-treated rats are not impaired in the active place avoidance task

The ipsilateral fimbria had a similar content as the contralateral fimbria 14 days after LPC injection suggesting remyelination (Figure 1). We therefore tested whether remyelination of the fimbria affected the behavior of the LPC-injected rats. Two groups of rats were injected with saline (n = 6) or LPC (n = 6) and returned to their home cage for 13 days. Beginning on the 13^th^ day, both groups received same hierarchy of behavioral tasks as the rats tested 4 days after injection. On open field testing both groups traveled a similar distance (Table 1). On passive place avoidance, both groups traveled a similar distance (F_(1,10)_  = 0.23, p>0.5). There was neither a significant effect of group (F_(1,10)_  = 1.08, p>0.3) nor trial (F_(1,30)_  = 1.70, p>0.1) for shock zone entrances. The lack of a significant trial effect likely resulted because saline- and LPC-injected groups rarely entered the shock zone in all four trials. The following day, both LPC- and saline-injected rats were tested on the active place avoidance task. On the 6^th^ trial of active place avoidance, there was no significant effect on average speed (F_(3,23)_  = 1.90, p = 0.16), linearity (F_(3,23)_  = 2.26, p = 0.11), or time to 1^st^ entrance (F_(3,23)_  = 0.88, p = 0.46).

Comparison of the learning curves of saline-injected rats at 4 and 14 days shows no significant effect of group (F_(1,10)_  = 0.20, p>0.5), but a significant effect of trial (F_(5,50)_  = 17.62, p<0.0001) with no interaction of group and trial (F_(5,50)_  = 1.64, p>0.1). These data show saline- and LPC-injected groups were able to lower the number of entrances, indicating equivalent learning of the shock zone location. 

Comparison of the entrances of rats injected with saline at 4 and 14 days showed a significant effect of trial (F_(5,50)_  = 2.51, p<0.05), but not of group (F_(1,10)_  = 3.09, p>0.05), with no interaction between group and trial (F_(5,50)_  =  0.78, p>0.5). These data suggest that task acquisition at days 4 and 14 did not significantly differ within saline rats. In contrast, rats injected with LPC showed a significant effect of both group (F_(1,10)_  = 5.05, p>0.05) and trial (F_(5,50)_  = 16.97, p<0.0001) with a significant interaction between group and trial (F_(5,50)_  = 7.6, p<0.001). Active place avoidance testing did show a significant effect of group on the number of shocks per entrance (F_(3,23)_  = 5.85, p<0.005; post-hoc, Saline 4D vs LPC 4D, p<0.005; LPC 4D vs. LPC 14D, p<0.01). These data strongly suggest that LPC-injected rats increased their ability to acquire the active place avoidance task between 4 and 14 days.

## Discussion

The major findings of this study are: (1) LPC injection into the fimbria produces a localized cycle of demyelination-remyelination, (2) four days after LPC injection, at a time when the fimbria is demyelinated, rats are impaired in the active place avoidance task and, (3) fourteen days after LPC injection, at a time when the fimbria has spontaneously remyelinated, rats are not impaired in the active place avoidance task.

Rats are impaired in the active place avoidance after complete cessation of activity in one hippocampus using tetrodotoxin [14]. Thus the acquisition of the active place avoidance requires the function of both hippocamppi. Four days after LPC injection, rats are impaired in acquisition of the active place avoidance (Figure 2). Impaired function of the fimbria leads to hippocampal dysfunction [24].

Demyelination of the commissural tracts in the fimbria by LPC interferes with salutatory transmission that either slows or blocks action potential between two hippocamppi. The effect of LPC-induced demyelination is suggested by what is known about hippocampal discharge after unilateral TTX injections. Unilateral TTX injection silenced the injected hippocampus while greatly increasing correlated firing of hippocampal principal cells in the uninjected hippocampus [15]. Thus, at times of LPC-induced fimbria demyelination, impaired interhippocampal informational flow is predicted to alter coordinated firing of pyramidal cell pairs within each hippocampus, perhaps by increasing co-activation. Correlated firing is hypothesized to decrease at times of remyelination of the fimbria. These predictions provide a framework to further study how behavioral dysfunction arises from impaired coordination of the two hippocamppi [15], [25].

Bilateral lesions of the retrosplenial cortex also produced deficits in the spaced version of the active place avoidance task [26]. The spaced version of the task has a 24-hour intertrial interval rather than the 10-minute intertrial interval used in this study. The severing of the corpus callosum or the ventral hippocampal commissure also prevented acquisition of the spaced version of the active place avoidance task (M. Wesierska, personal communication). Thus, acquisition of the active place avoidance task appears to need communication between the hippocamppi or the cerebral cortices. The finding that LPC- injections into the fimbria produced deficits in acquiring the active place avoidance task suggests that LPC-injections into the corpus callosum may produce similar deficits.

Four days after injection, LPC-injected rats had significantly more shocks per entrance than saline-treated rats even though LPC- and saline-injected rats needed a similar shock intensity to leave the shock zone (Table 1). Thus, the increased in shocks per entrances at 4 days post-injection was not due to differences in the ability of the rat to feel shock and induce escape behavior. The increased shocks per entrance in LPC-injected rats were no longer seen 14 days after injection. In addition to commissural white matter tracts, the fimbria contains tracts projecting to other brain regions via the fornix [22]. Lidocaine was anxiogenic when injected into the post-commissural fimbria [27]. LPC-injected rats tended to freeze when receiving shocks during active place avoidance resulting in the increased shocks per entrance as seen in altered tendencies of less distance traveled, lower speed and higher linearity than saline-treated rats (Table 1). The increase in shocks per entrance is no longer seen 14 days after LPC injection suggesting a role for remyelination of the fimbria.

Rats injured in the moderate form of the CCI model of TBI had similar impairment in the active avoidance task as rats having LPC induced demyelination of the fimbria. Unlike LPC injection, moderate CCI produces widespread white matter damage that includes the fimbria, the corpus callosum and dorsal hippocampal commissure, as well as grey matter loss in the cortex [17]. This study demonstrates that demyelination of the fimbria is sufficient to impair acquisition of the active place avoidance task. This suggests that the active place avoidance is sensitive to white matter injury. Minocycline improved acquisition of the active avoidance task while N-acetylcysteine, progesterone, simvastatin and cyclosporine did not [17]. Interestingly, minocycline alone was able to increase myelin content in multiple white matter tracts after moderate CCI [17]. Drug testing using the outcome of the improved acquisition of active place avoidance task selected a drug that acted on myelin. The active place avoidance task may also be valuable to find additional drugs that either prevent or repair white matter damage following traumatic brain injury.
